# Accessory Renal Artery Stenosis and Secondary Hypertension

**DOI:** 10.1155/2020/8879165

**Published:** 2020-07-22

**Authors:** Ariel A. Chung, Patricia R. Millner

**Affiliations:** Department of Family Medicine, Tripler Army Medical Center, Honolulu, HI 96859, USA

## Abstract

**Background:**

Secondary hypertension is an uncommon cause of hypertension with extensive workup not recommended in most patients; however, further evaluation is generally recommended in young patients presenting with hypertension. *Case Presentation.* A 31-year-old female presented with history of elevated blood pressures. Secondary hypertension workup revealed no laboratory abnormalities; however, renal artery ultrasound demonstrated a left superior accessory artery and suspected bilateral renal vein congestion that was further evaluated with renal CT with contrast. Renal CT showed ostial stenosis of the left accessory renal artery. In addition, compression of the left renal vein between aorta and superior mesenteric artery was also noted, consistent with nutcracker syndrome. Hypertension was suspected to be secondary to stenosis of the accessory renal artery. Upon consultation with interventional radiology, pharmacologic treatment was recommended, and blood pressure control was ultimately achieved with a single agent. *Discussion.* Renovascular etiologies are responsible for 1% of cases of mild hypertension and up to 45% of severe hypertension. Accessory renal arteries are a normal anatomical variant in approximately 30% of the population. Secondary hypertension due to stenosis of an accessory renal artery is rare with very few cases described in case reports.

**Conclusion:**

Though hypertension secondary to accessory renal artery stenosis is rare and not well published in medical literature, few case reports, including this one, demonstrate that accessory renal artery stenosis can be an underlying etiology of hypertension.

## 1. Background

Hypertension is a common diagnosis in the United States, affecting approximately 30% of adults; however, secondary hypertension is uncommon and responsible for only 5–10% of cases [1, 2]. Hypertension due to secondary causes is more common in the younger population; however, it can occur in all ages [2]. The most common etiologies of secondary hypertension vary by age and include renal parenchymal disease, renal artery stenosis, coarctation of the aorta, thyroid dysfunction, hyperaldosteronism, obstructive sleep apnea, Cushing's syndrome, and pheochromocytoma [2]. Evaluation for secondary causes of hypertension is generally not recommended in all individuals presenting with elevated blood pressure but should be considered in patients with severe or resistant hypertension, presence of end organ damage, or onset before 30 years of age [2].

## 2. Case Presentation

A 31-year-old female presented for a routine clinic visit with her primary care physician and was found to have an elevated blood pressure of 150/100 that was sustained on repeat measurement. Upon further questioning, the patient reported history of elevated blood pressure readings over the past 7 years and was lost to follow-up. Past medical history was significant for migraine headaches and the patient had no past surgical history. Current medications included rizatriptan and ibuprofen were used as needed for headaches, approximately 2–3 times per year, and levonorgestrel IUD for contraception. Family history was notable for maternal and paternal grandparents with hypertension and mother with hypothyroidism. The patient was a nonsmoker with occasional alcohol use. BMI was 19. Physical exam was unremarkable.

**Figure 1:**
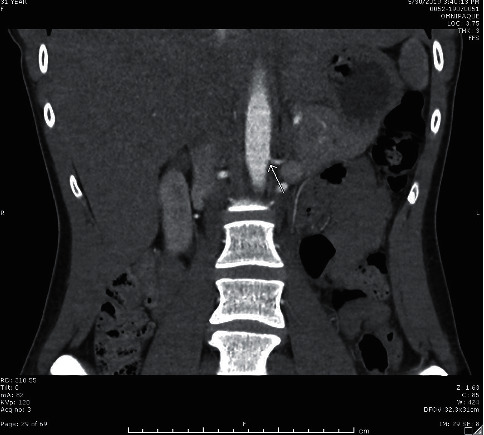
Coronal image from renal CT with contrast showing the area of ostial stenosis of left accessory renal artery.

**Figure 2:**
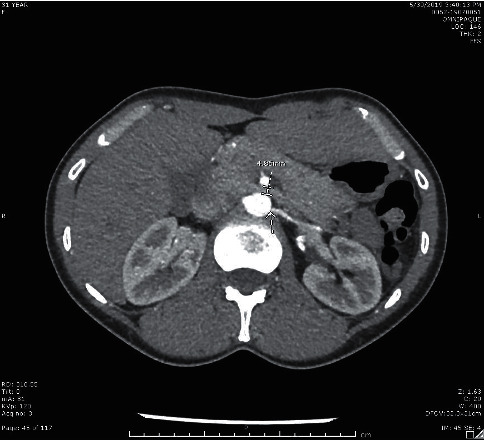
Axial image from renal CT with contrast showing the area of ostial stenosis of left accessory renal artery and compression of left renal vein between aorta and superior mesenteric artery.

**Figure 3:**
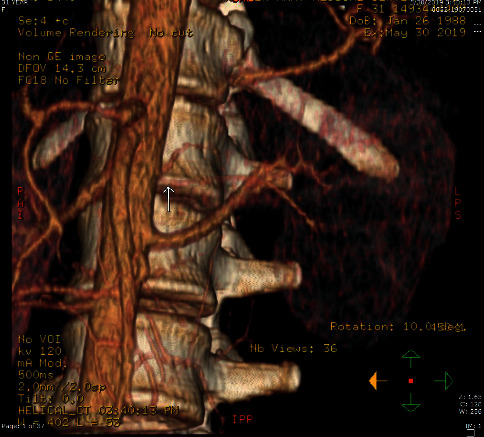
3D image from renal CT with contrast showing the area of ostial stenosis of left accessory renal artery.

**Figure 4:**
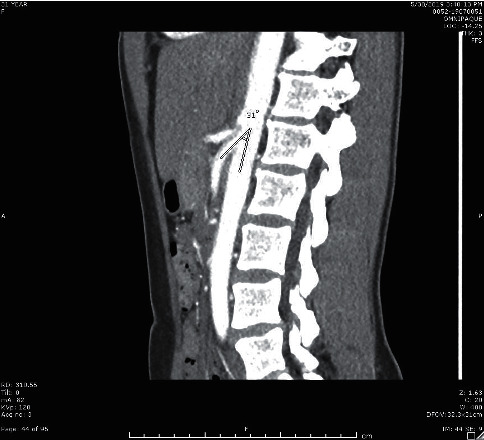
Sagittal image from renal CT with contrast showing decreased angle between aorta and superior mesenteric artery.

24-hour ambulatory blood pressure monitoring was performed and confirmed the diagnosis of hypertension. Due to the patient's age, a secondary hypertension workup was initiated. Laboratory evaluation included complete blood count, basic metabolic panel, and thyrotropin that showed no abnormalities; plasma aldosterone and renin were within normal limits at 2.9 and 1.069, respectively; urinalysis showed 1+ protein with protein/creatinine ratio 0.44. Renal ultrasound was performed and showed presence of left accessory renal artery and the appearance of bilateral renal vein congestion. Bilateral main renal arteries showed no signs of stenosis. Renal CT with contrast was recommended for further evaluation of renal vasculature due to appearance of renal vein congestion and showed ostial stenosis of the left accessory renal artery (Figures 1–3). In addition, compression of the left renal vein between aorta and superior mesenteric artery was also noted, consistent with nutcracker syndrome (Figures 2 and 4).

The cause of hypertension in this 31-year-old female was suspected to be due to stenosis of the left accessory renal artery that was identified on imaging. Interventional radiology was consulted and recommended trial of pharmacologic treatment before pursuing angiography or revascularization. An antihypertensive regimen was initiated with nifedipine extended release 30 mg that was discontinued due to side effects of headaches and lower extremity edema. Nifedipine was replaced with amlodipine 10 mg; however, amlodipine at maximum dose did not achieve desired blood pressure goal. Lisinopril 5 mg was added to the regimen at this time with subsequent improvement of blood pressure; however, the patient experienced several episodes of presyncope likely due to orthostatic hypotension. Amlodipine was discontinued and lisinopril dose was increased to 10 mg and desired blood pressure goal was achieved without significant side effects.

## 3. Discussion

Renovascular hypertension is responsible for approximately 1% of cases of mild hypertension and approximately 10–45% of severe cases [3, 4]. The two main etiologies of renal artery stenosis are atherosclerosis and fibromuscular dysplasia [4]. Hypertension due to renal artery stenosis is the result of activation of the renin-angiotensin-aldosterone system due to renal ischemia [4]. The standard for diagnosis is invasive angiography; however, ultrasonography, magnetic resonance angiography (MRA), and CT angiography (CTA) are preferred as noninvasive initial studies [4]. Potential treatment options include pharmacologic as well as revascularization, with revascularization typically reserved for patients with hypertension refractory to antihypertensive medications or progressive worsening of renal function [4].

Accessory renal arteries are a normal anatomic variant in approximately 30% of the population [5]. Hypertension due to stenosis of an accessory renal artery in the absence of stenosis of the main renal arteries is exceedingly rare, with very few cases described in case reports. To the best of our knowledge, there are only two case reports that exist in published literature that describe patients with elevated blood pressure and secondary hypertension workup notable for stenosis of an accessory renal artery.

Zeina et al. describe a case of a 35-year-old female with resistant hypertension despite regimen with multiple antihypertensive agents [6]. Invasive angiography was performed and showed stenosis of an accessory renal artery with an appearance consistent with fibromuscular dysplasia [6]. Revascularization with balloon angioplasty was performed with subsequent improvement in blood pressure and the ability to achieve desired blood pressure goal with a single antihypertensive agent [6].

Akbeyaz et al. describe a case of a 13-year-old female with hypertension secondary to stenosis of an accessory renal artery identified on renal ultrasound and confirmed on CT angiography [7]. The patient received antihypertensive pharmacologic treatment and blood pressure goal was achieved [7]. At routine follow-up two months later, it was discovered that the patient's blood pressure was much lower than previous measurements and antihypertensive treatment was gradually tapered and discontinued [7]. Repeat renal ultrasound was performed and the accessory artery could no longer be visualized [7]. Spontaneous collapse of the vessel was suspected [7]. The patient remained normotensive and did not require any additional treatment [7].

In our case, the patient was also a young female with hypertension suspected to be due to the accessory renal artery stenosis that was discovered on imaging. In contrast, our patient received renal CT with contrast. This imaging modality is not typically recommended to evaluate stenosis of renal arteries; however, this study was recommended to further evaluate bilateral renal vein congestion that was seen on renal ultrasound and incidentally discovered stenosis of the left accessory renal artery. Imaging also revealed compression of the left renal vein between aorta and superior mesenteric artery consistent with nutcracker syndrome. We considered nutcracker syndrome as a possible etiology of the patient's hypertension due to a small number of published case reports that suggested that hypertension may be a rare symptom of nutcracker syndrome [8–11]. However, in the vast majority of these cases, the patients presented with additional symptoms that are commonly associated with nutcracker syndrome including flank pain and hematuria [8–11]. Due to the absence of additional symptoms in our patient, we suggest that stenosis of the accessory renal artery is the most likely etiology of the patient's hypertension. Our patient's hypertension was ultimately controlled on one antihypertensive agent and did not require further invasive testing or intervention.

## 4. Conclusion

Though hypertension secondary to accessory renal artery stenosis is rare and not well published in medical literature, few case reports, including this one, demonstrate that accessory renal artery stenosis can be an underlying etiology of hypertension.

## Data Availability

Information regarding this case is available on the electronic medical record of Tripler Army Medical Center.
